# Anterior Cervical Discectomy and Fusion: A Hidden Etiology of Obstructive Sleep Apnea

**DOI:** 10.7759/cureus.22185

**Published:** 2022-02-13

**Authors:** Waiz Wasey, Sharefi Saleh, Naila Manahil, Asiya Mohammed, Neha Wasey

**Affiliations:** 1 Family and Community Medicine, Southern Illinois University (SIU) School of Medicine, Springfield, USA; 2 Family Medicine, Ruth Temple Clinic, Los Angeles, USA; 3 Family Medicine, Southern Illinois University (SIU) School of Medicine, Springfield, USA; 4 Family Medicine, Southern Illinois University (SIU) Center for Family and Community Medicine, Springfield, USA; 5 General Practice, Shadan Institute of Medical Sciences, Hyderabad, IND

**Keywords:** automatic positive airway pressure, obstructive sleep apnea, anterior cervical discectomy and fusion, cervical spine surgery, apap, cpap, osa, acdf

## Abstract

Obstructive Sleep Apnea (OSA), a common variant of sleep-disordered breathing, is characterized by repeated complete or partial collapse of upper airways during sleep, leading to oxyhemoglobin desaturations. The obstruction may be iatrogenically induced in patients undergoing Anterior Cervical Discectomy and Fusion (ACDF). Damage to the pharyngeal plexus during the procedure may predispose to a new collapse of the upper airway, and the placement of the hardware for the fusion may lead to further narrowing of the upper airway. Literature does not exist associating this possible etiology prospectively. The association of ACDF and OSA has only been retrospectively documented by Guiilleminault and associates. We are reporting a case of a 49-year-old female who was evaluated pre and post surgery and established the evidence of worsening OSA in a patient who underwent ACDF.

## Introduction

Obstructive sleep apnea (OSA) is a breathing disorder characterized by recurrent episodes of upper airway collapse and obstruction during sleep leading to oxygen desaturations. These episodes may or may not be associated with arousals from sleep [1]. The recurrent desaturations, arousals, or both lead to fragmented sleep and daytime functional impairment. Risk factors of OSA have been documented thoroughly in medical literature as modifiable and nonmodifiable factors. These include, but are not limited to obesity, age, sex, family history, retrognathia, heart disease, stroke, diabetes, polycystic ovarian syndrome, acromegaly, and Down syndrome [2].

Anterior cervical discectomy and fusion (ACDF) is one of the most common cervical spine procedures done in the United States (US). It is estimated that about 137,000 ACDF procedures are performed annually [3]. The morbidity rates from ACDF are documented to range from 13.2-19.3%. Dysphagia alone accounts for 9.5% [4]. The etiology of dysphagia is likely from the damage to the pharyngeal nerve or pharyngeal plexus in the neck. The placement of hardware in the cervical area for the fusion along with dysfunction of the pharyngeal plexus is hypothesized to lead to narrowing and collapse of the upper airway [5]. This iatrogenic association of ACDF with OSA has been poorly studied. Only one retrospective study done in 2003 by Guilleminault and associates hinted at the prevalence of OSA in patients who underwent ACDF [6].

Our case report prospectively follows a patient undergoing ACDF and establishes a diagnosis of OSA post-surgery. The aim of this report is to encourage more prospective data to highlight the prevalence of OSA in this patient population. As ACDF is a common procedure, timely diagnosis and treatment of OSA could improve overall morbidity and mortality.

## Case presentation

A 49-year-old female with a past medical history of cervical myelopathy was evaluated in the sleep clinic for snoring, witnessed apnea, and daytime somnolence. She scored 11 on the Epworth Sleepiness Scale (ESS). On examination, she had a narrow airway with a Mallampati score of 4. Her symptoms and physical examination indicated the presence of sleep-disordered breathing (SDB). She slept seven to eight hours consistently and reported no symptoms suggestive of hypersomnia. To further evaluate the SDB, diagnostic testing was ordered. 

A home diagnostic sleep test revealed mild OSA with an apnea-hypopnea index (AHI) of 6.8/hr (Table 1). Alice NightOne home sleep testing (HST) device (Koninklijke Philips N.V., Amsterdam, Netherlands) was used for this study.

**Table 1:** Home Sleep Study performed prior to ACDF AHI: apnea-hypopnea index; ACDF: anterior cervical discectomy and fusion

Diagnostic Parameters	Results
Total Sleep Time	338 mins
AHI	6.8/hr
Lowest Oxygen (O2) Saturation	84%
Total time of O2 <89%	5 mins

**Figure 1:**
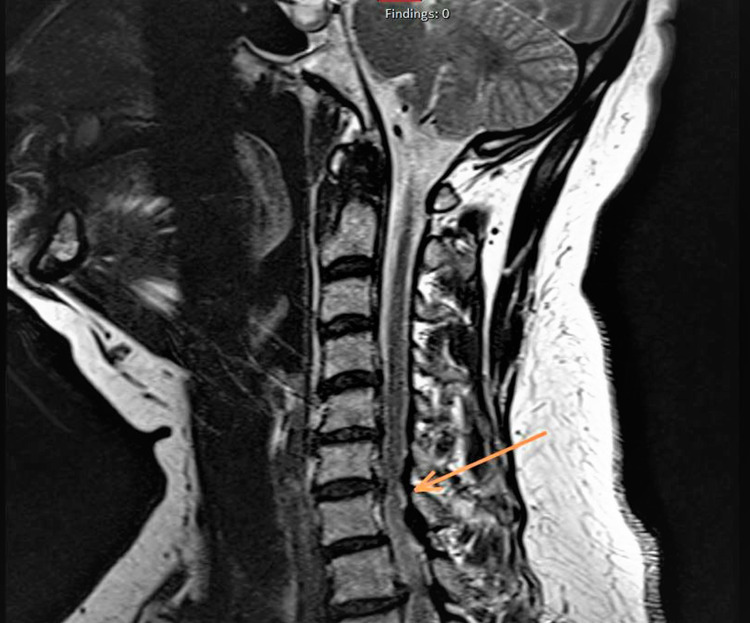
Cervical MRI showing multi-level disc herniation and foraminal canal stenosis at C5-C6 and C6-C7 spine

Based on the results of the sleep study, she was started on an Auto Continuous Positive Airway Pressure (APAP). She struggled with using the device due to claustrophobia. The limited compliance data that was available from APAP showed an improved AHI of 3.3 with use. Desensitization methods were initiated to help with compliance and increased use of the APAP. In the interim, the patient's cervical symptoms worsened (Figure 1). The pain led to decreased use of APAP and total sleep time.

**Figure 2:**
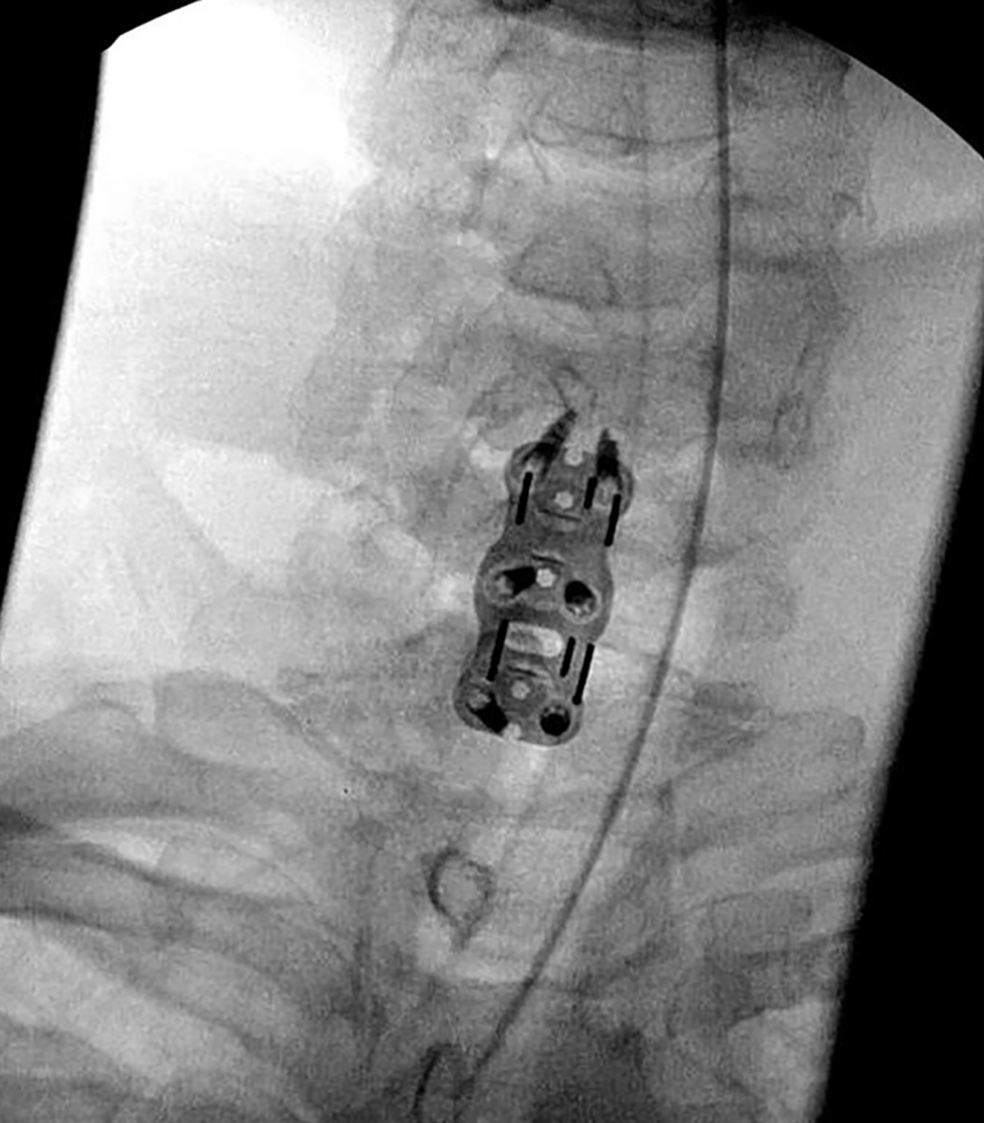
X-ray cervical spine post-ACDF plate placement ACDF: anterior cervical discectomy and fusion

Roughly six months after her first evaluation in the sleep clinic, she underwent ACDF (Figure 2). The procedure was complicated with post-surgical infection and the patient was admitted to the hospital for intravenous antibiotics.

During her clinical course in the hospital, significant oxygen desaturations to 80% were noted during sleep. The patient also reported awakening from choking and snoring at night. She reported mild dysphagia as well and was evaluated with fluoroscopic studies. These symptoms continued even after the resolution of the post-surgical infection. She was referred back to the sleep clinic for re-evaluation six weeks after surgery.

The patient reported more snoring, frequent awakenings at night, and nonrestorative sleep. She had lost 20 pounds since her first sleep study and her BMI improved to 33.99 kg/m^2^ from 37.35 kg/m^2^. Except for the addition of hydrocodone 5mg with acetaminophen 325, once or twice daily as needed for pain, no other medication changes were made during the hospital stay. A new diagnostic home sleep study test was ordered to re-evaluate her symptoms. The repeat HST showed progression of her OSA to moderate severity with an AHI of 24.5/hr (Table 2). WatchPAT™ home sleep apnea device (IItamar Medical Ltd, Caesarea, Israel) was used for the repeat study.

**Table 2:** Home sleep study post-ACDF AHI: apnea-hypopnea index; ACDF: anterior cervical discectomy and fusion

Sleep Parameters	Results
Total Sleep Time	212 mins
AHI	24.5/hr
Rapid Eye Movement (REM) AHI	46.6/hr
Lowest O2 saturation	90%
Time of O2 <89%	0 mins

**Figure 3:**
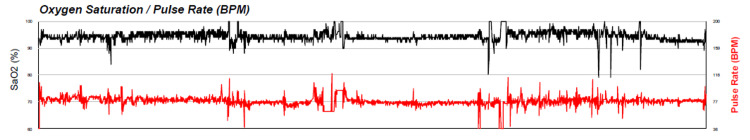
Oxygen tracing on the second home sleep study

As the sleep study did not demonstrate central sleep apnea or hypoventilation (Figure 3), it was unlikely that the worsened AHI was due to the opioid medication. It was concluded that the ACDF procedure led to worsened OSA.

## Discussion

OSA is characterized by recurrent episodes of upper airway collapse and obstruction during sleep, leading to oxygen desaturations. These episodes may or may not be associated with arousals [1]. The recurrent desaturations or arousals or both, lead to fragmented sleep. The severity and impact of OSA on health vary based on risk factors. These risk factors have been classified in medical literature as modifiable and unmodifiable. The unmodifiable factors are inherited as anatomical structural abnormalities. These include a genetically inherited narrow airway, craniofacial deformities, micro- or retrognathia, and inferior positioning of the hyoid bone [7]. The modifiable risk factors include age, weight, medication use, substance abuse, underlying cardiac, pulmonary, or endocrinological disease, as well as nasal obstruction. Theoretically, any surgical procedure that alters the upper airway can influence OSA, as exemplified by our case study. 

ACDF is one of the most commonly performed cervical spine operations, with an estimate of 137,000 procedures per year in the US alone [3]. The surgery is performed with an aim to relieve neuropathic symptoms as a result of cervical spine disc herniation and narrowing of the spinal canal. The morbidity that arises from this widely performed surgery is estimated to be 13.2-19.3%, with the most common and immediate complication being dysphagia [4]. The underlying cause for dysphagia is multifactorial, and one of them includes injuries to the pharyngeal plexus [8]. This may explain the mechanism leading to OSA as well. The dysfunction of the pharyngeal plexus along with narrowing of the airspace post plate placement in the cervical area leads to collapse and narrowing of the airway, hence development or a possible worsening of OSA. Medical literature outlying the complications post ACDF does not mention OSA, nor is there any sleep literature associating the two. The only literature found on OSA and spinal fusion was published in 2003 by Guilleminault et al, in which they reported a retrospective association between OSA and ACDF in 12 patients [6]. 

Our patient had been evaluated months prior to the procedure and was found to have mild OSA. This was most likely due to her obesity as well as inherited narrow airways as she had a Mallampati score of 4. Post-surgical evaluation with sleep study revealed moderate OSA. We utilized HST for both evaluations. HST is convenient to schedule and administer, as well as are affordable. The parameters studied by HST include oxygen flow, oxyhemoglobin levels, breathing effort, heart rate, and sleeping positions. These parameters are adequate to diagnose someone with OSA. In the laboratory, polysomnography has added channels to evaluate electroencephalogram (EEG) patterns, muscle tone, and limb movements. We did not require these parameters for evaluating the upper airway effects of ACDF and hence resorted to HST. 

While trying to decipher the underlying cause of her worsening OSA, it was noted that her weight had improved by 20 pounds. Various studies have shown improved severity of OSA with weight loss [9]. The only medication change was the addition of opioids for pain control. Opioids are known to cause hypoventilation and central sleep apnea (CSA) more than OSA [10], but these were not observed in the repeated sleep study. In fact, her oxygen saturation remained above 90% as compared to her original study. This concluded that the only explanation for the worsening of OSA was the post-surgical effect of ACDF.

## Conclusions

OSA is a common sleep breathing disorder, and ACDF is a widely common cervical surgical procedure. Although not strongly associated in medical literature, ACDF may precipitate the onset of OSA, or worsen an already existing one. Prospectively following our patient clearly demonstrates the strong association of ACDF. Our report highlights the need for prospective studies in this cohort to establish a strong association between the two, as untreated OSA is known to cause morbidity. Timely diagnosis and treatment of OSA in patients undergoing ACDF will help prevent morbidity, as a result of better sleep quality. 
